# Dietary Supplementation with Sea Bass (*Lateolabrax maculatus*) Ameliorates Ulcerative Colitis and Inflammation in Macrophages through Inhibiting Toll-Like Receptor 4-Linked Pathways

**DOI:** 10.3390/ijms20122907

**Published:** 2019-06-14

**Authors:** Jiali Chen, Muthukumaran Jayachandran, Wenxia Zhang, Lingyuqing Chen, Bin Du, Zhiling Yu, Baojun Xu

**Affiliations:** 1 Programme of Food Science and Technology, Division of Science and Technology, Beijing Normal University-Hong Kong Baptist University United International College, Zhuhai 519087, China; kellychan123@126.com (J.C.); jmkbio@uic.edu.hk (M.J.); l630013050@mail.uic.edu.hk (W.Z.); j430013005@mail.uic.edu.hk (L.C.); 2 Centre for Cancer and Inflammation Research, School of Chinese Medicine, Hong Kong Baptist University, Hong Kong, China; 3 Hebei Key Laboratory of Natural Products Activity Components and Function, Hebei Normal University of Science and Technology, Qinhuangdao 066004, China; bindufood@aliyun.com

**Keywords:** inflammation, ulcerative colitis, dietary therapy, TLR4 signaling

## Abstract

Sea bass (*Lateolabrax maculatus*) is a kind of food material commonly consumed in daily life. In traditional Chinese medicinal books, it has been indicated that sea bass can be applied for managing many inflammation-associated conditions. However, the studies on the pharmacological mechanisms of inflammation of sea bass remain scarce. Hence, this study aims to investigate the molecular mechanisms of the anti-inflammatory activity of sea bass. Anti-inflammatory activities of sea bass were assessed using dextran sulfate sodium (DSS)-induced colitis in a mice model and lipopolysaccharide (LPS)-activated macrophages model. Low body weight and short colon length were observed in DSS-fed mice that were significantly recovered upon sea bass treatments. Moreover, the colon histopathology score showed that sea bass-treated mice had decreased crypt damage, focal inflammation infiltration and the extent of inflammation, suggesting that treatment with sea bass could attenuate intestinal inflammation. In addition, the in-vitro study conjointly indicated that sea bass could suppress the inflammatory mediators in LPS-activated macrophage by inhibiting the TLR4-linked pathway. The present findings demonstrated that sea bass has an inhibitory effect on TLR4 signaling; thus, it could be a promising candidate for treating inflammation-associated conditions. A further justification for the clinical application of sea bass in treating inflammation-associated conditions is necessary.

## 1. Introduction

Inflammatory bowel disease (IBD) is principally outlined as Crohn’s disease and ulcerative colitis (UC). Crohn’s disease might have an effect on any part of the digestive tract, from the mouth to the anus with diarrhea and abdominal pain. Ulcerative colitis (UC) mainly presents in the rectal and colonic mucosa and is accompanied by weight loss, diarrhea, abdominal pain, and rectal bleeding. This kind of uncontrolled gut inflammation affects millions of individuals in the world [1,2]. It has been reported that the worldwide incidence and prevalence of UC are increasing, especially in newly industrialized countries. The highest reported prevalence values appeared in Europe and North America [1,2]. In population-based studies, it also indicated that UC patients will have proximal disease extension within 10 years [3]. In the 21st century, systematic research into UC prevention and dietary therapy development seems very significant with an irresistible trend. However, the pathogenesis of IBD remains unclear due to lack of investigation. IBD is a multifactorial disorder induced by the interaction of genetic factors, environment, microbiota, and immune response, which are involved in pathogenesis [4,5]. Recently, many studies have indicated that the breakdown of homeostasis among the immune system, epithelial barrier, and gut microbiome might be the critical underlying mechanism responsible for the development of IBD [4,5]. Previous reports suggested that the products of gut microbiota could positively have an effect on the pathogenesis of inflammation-associated diseases [6,7]. The gastrointestinal tract provides residence to both beneficial and potentially pathogenic microorganisms. The imbalance within the microbiota composition may worsen the dysbiosis in the inflamed gut [6]. Due to the immune modulatory role of gut microbiota, sea bass is hypothesized to have beneficial effects on the host immune response and amelioration of intestinal inflammation. Moreover, the dextran sulfate sodium (DSS) model resembles UC in several pathophysiological and morphological features, including the production of pro-inflammatory cytokines, crypt damage, focal inflammation infiltration, and ulceration [8]. Generally, colitis is induced chemically in this model by adding DSS to the drinking water of mice. It mainly affects the distal colon; some inflammatory responses appeared even in the proximal colon and caecum. The outcome of this model may be affected by the genetic background of the animals, environment, and the DSS concentration [6,8]. Additionally, body weight, feed consumption, and colon length were treated as an indication of the disease severity in the DSS-induced model [9,10,11]. Therefore, DSS-induced colitis model is employed to study the efficacy of aqueous extract of sea bass (ASB) in managing inflammation-associated conditions (colitis). Toll-like receptor 4 (TLR4) signaling is one of the important mechanisms for inflammation-related studies and it is a key receptor for commensal recognition in gut innate immunity [12]. It was the subject of target inhibition in ulcerative colitis (UC) [12,13]. Moreover, AP-1 and NF-κB were treated as the critical and classical pathways in TLR4 signaling. Many researchers have already elucidated that over-expression of TLR4-linked AP-1 or NF-κB is typical in inflamed colonic tissue [13,14]. Therefore, it is necessary to evaluate the effects of TLR4 signaling in DSS-induced colitis for studying UC in detail.

Sea bass (*Lateolabrax maculatus*) is an economically important cultured fish species and has a long history of managing inflammation-associated conditions. However, the mechanism of action of sea bass needs to be investigated. Therefore, the aim of this study was to study the efficacy of ASB in managing inflammation-associated conditions by in vitro and in vivo experiments. The DSS-induced colitis model was used for discovering ulcerative colitis in vivo. Many studies have reported that the process of colitis is closely linked with neutrophils and macrophages [14,15,16]. It is well known that macrophages play vital roles in innate immunity for the inhibition of inflammatory cytokines [17,18]. As one of the typical in vitro models for investigating inflammation, lipopolysaccharide (LPS)-activated macrophages were used in this study [17,19,20]. Moreover, many reports have indicated that inflammation in macrophages is closely linked to the activation of TLR4 signaling [21,22,23]. In addition, NF-ĸB and AP-1 are the typical pathways in TLR4 signaling which are associated with the inflammation triggered by the innate immune system [24,25]. The activation of NF-ĸB or AP-1 pathways will lead to the production of a series of inflammatory mediators, including interferon gamma (IFN-γ), tumor necrosis factor alpha (TNF-α), and monocyte chemoattractant protein-1 (MCP-1). The TNF-α is a pleiotropic cytokine, which is an important mediator of inflammation [26]. The inhibition of TNF-α secretion in LPS-induced macrophages results in anti-inflammation [27]. MCP-1 is a highly representative chemokine, critical for the pathogenesis of liver disease and granulomatous inflammation [28,29]. The regulation of TNF-α, MCP-1, and IFN-γ via NF-κB, and AP-1 pathways are important mechanisms in inflammatory responses [28]. The therapeutic potential of sea bass against ulcerative colitis has not yet been discovered. Therefore, a good understanding of the anti-inflammatory activities of sea bass is essential for providing the pharmacological basis for the folk use of sea bass, and further, its application in the medical industry. 

## 2. Results

### 2.1. Characterization of the Aqueous Extract of Sea Bass (ASB)

**Figure 1:**
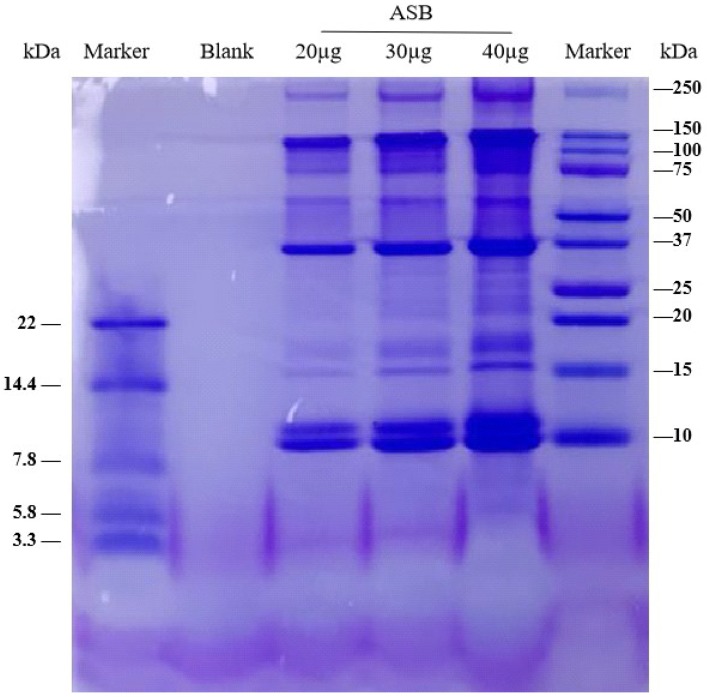
SDS-PAGE images of the aqueous extract of sea bass (ASB).

To characterize the aqueous extract of sea bass, the crude protein content of ASB, the molecular weight of the composition of protein fractions in ASB, and the composition of amino acids in ASB were evaluated. Results showed that the crude protein value of ASB ranged from 74.91% to 78.87%. As shown in Figure 1, the molecular weight of protein fractions in ASB ranged from 3.3 to 250 kDa. In particular, the molecular weight of ASB protein fractions was distributed around 150, 37, and 10 kDa, respectively. As shown in Supplementary Figures S1 and S2, amino acids in ASB were quantified and identified in the chromatogram.

### 2.2. ASB Ameliorated DSS-Induced Colitis

**Figure 2:**
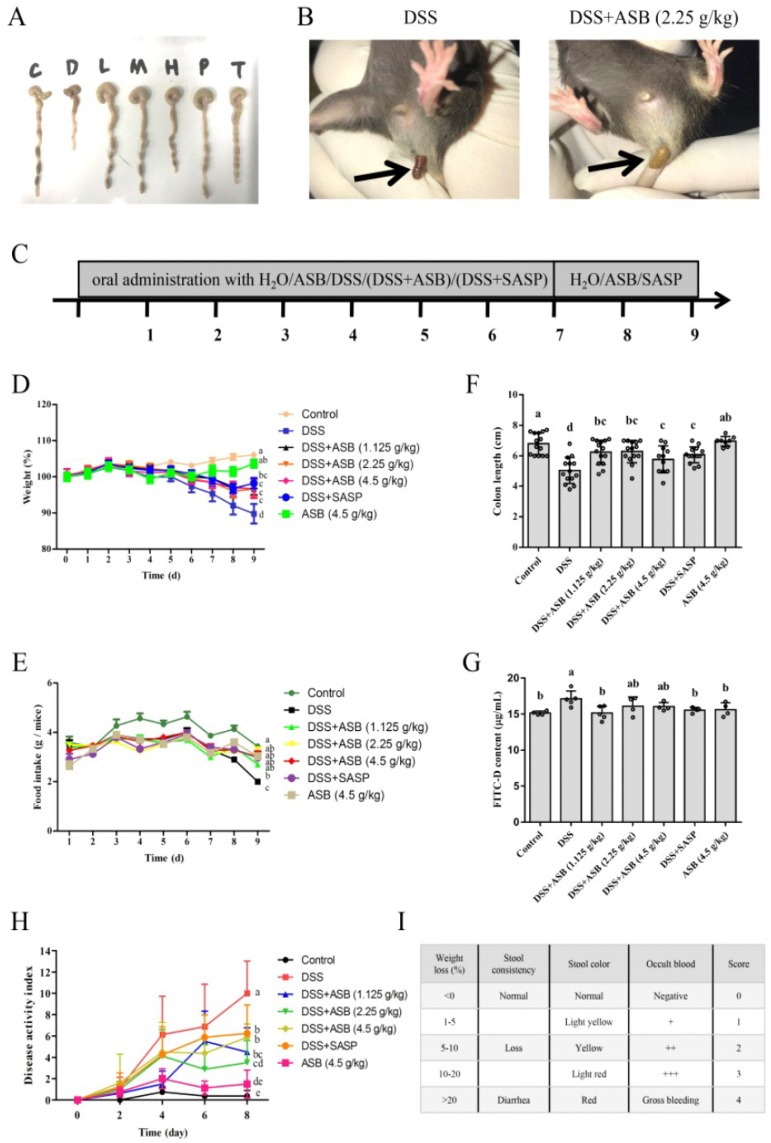
ASB protects against DSS-induced colitis in mice. (**A**) Representative photographs of colon (C: control, *n* = 14; D: DSS model, *n* = 14; L: DSS+ASB low dosage, *n* = 14; M: DSS+ASB medium dosage, n=14; H: DSS+ASB high dosage, *n* = 14; P: SASP, *n* = 14; T: ASB high dosage reference, *n* = 8). (**B**) Effect of ASB on rectal bleeding in DSS-treated mice at day 7. (**C**) Schematic representations of the colitis model. Effect of ASB on body weight (**D**), daily feed content (**E**) and colon length (**F**) at the end of the experiment. (**G**) Effect of intestinal permeability upon ASB treatments at the end of the experiment. (**H**) Disease activity index (DAI) evaluation of mice in each group. (**I**) The scoring criteria for DAI. Results were expressed as mean ± SD. Parameters marked by the same letter are not significantly different. Significance is represented as *p* < 0.05.

DSS-induced colitis model was constructed to explore the role of ASB in UC. After DSS feeding, a significant body weight loss, less fodder consumption, and bloody stools were observed, especially in the DSS group. As shown in Figure 2D, mice treated with ASB or sulfasalazine (SASP) showed body weight recovery compared to the DSS-treated group. Mice body weight did not show any marked changes in the high dosage reference group, which was similar to the control group. In accordance with the results shown in Figure 2D, DAI (Figure 2H) also indicated that mice treated with ASB or SASP could ameliorate the severity of colitis, compared to the DSS-treated group. Meanwhile, the amount of feed consumption also demonstrated a significant improvement upon ASB or SASP treatments in comparison with the DSS-treated group (Figure 2E). As shown in Figure 2A,F, DSS-induced colitis caused a marked decrease in colon length, while it improved upon ASB or SASP treatment. No obvious colon length change was observed in the high dosage reference group, compared to the control group. As is evident from the results, DSS-induced colitis in mice was ameliorated upon ASB treatments at the dosage of 1.125, 2.25, and 4.5 g/kg b.w. (the human equivalent dose (HED): 200 g/60 kg, 400 g/60 kg, 800 g/60 kg, respectively), which was similar to the treatment with SASP. 

### 2.3. Hematological Parameters

As shown in Table 1, mice with DSS administration showed significant (*p* < 0.05) anemia (lower red blood cell (RBC) levels, hemoglobin (HGB), hematocrit (HCT), mean corpuscular volume (MCV), and platelet distribution width (PDW)) in comparison with the control group. Meanwhile, the results also showed that supplementation of the diet with ASB could ameliorate these symptoms. Significant differences (*p* < 0.05) were found in the RBC levels, HCT, and PDW upon low dosage ASB treatment in comparison with the DSS model group. 

### 2.4. ASB Reduced Intestinal Permeability

Increase in gut permeability was linked with greater susceptibility to colitis. As shown in Figure 2G, the permeability of FITC-Dextran was significantly increased in the DSS-treated group, while such a change was improved upon ASB or SASP treatment. The high dosage reference group did not demonstrate a significant increase in comparison with the control group.

### 2.5. ASB Reduced Colonic Tissue Damage

**Figure 3:**
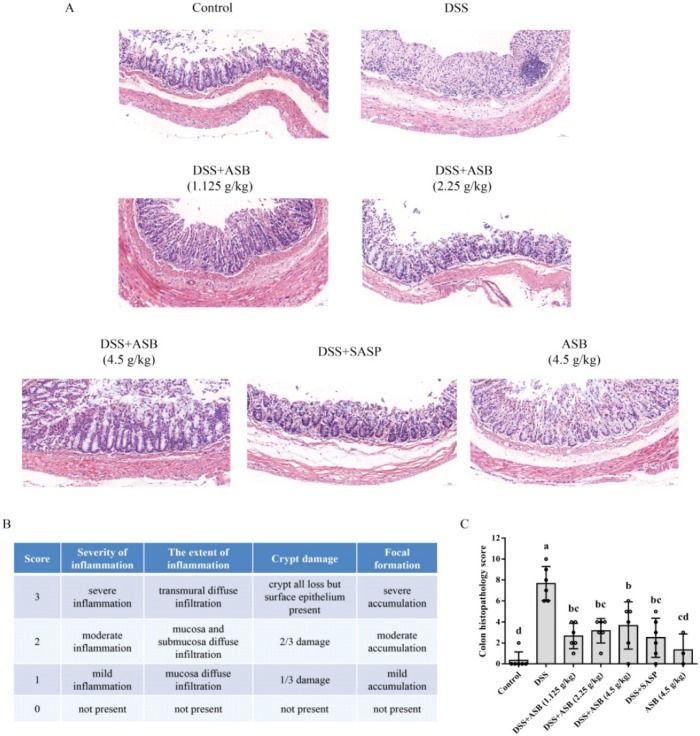
Effect of ASB on histopathological changes of mice in DSS-induced colitis. (**A**) Histological analysis (scale bar: 50 µm); (**B**) chart indicating scoring criteria for the evaluation of intestinal inflammation; (**C**) histological score. Results were expressed as mean ± SD (*n* = 3~6). Colon histopathology score marked by the same letter is not significantly different. Significance is represented as *p* < 0.05.

As shown in Figure 3A, 1.5% DSS in drinking water resulted in extensive colonic tissue damage, including inflammatory cell infiltration, crypt damage, and focal formation. Results showed that less colonic tissue damage was presented upon ASB treatments, as compared to the DSS-treated group. Diffuse infiltration of inflammation in mucosa and submucosa and crypt damage in colonic tissue was markedly increased in the DSS group, while such changes were significantly suppressed in the ASB-treated groups (Figure 3A,B). Meanwhile, mice in the high dosage reference group presented normal, similar to the control group.

### 2.6. ASB Inhibited the Neutrophil Infiltration in Impaired Colon

**Figure 4:**
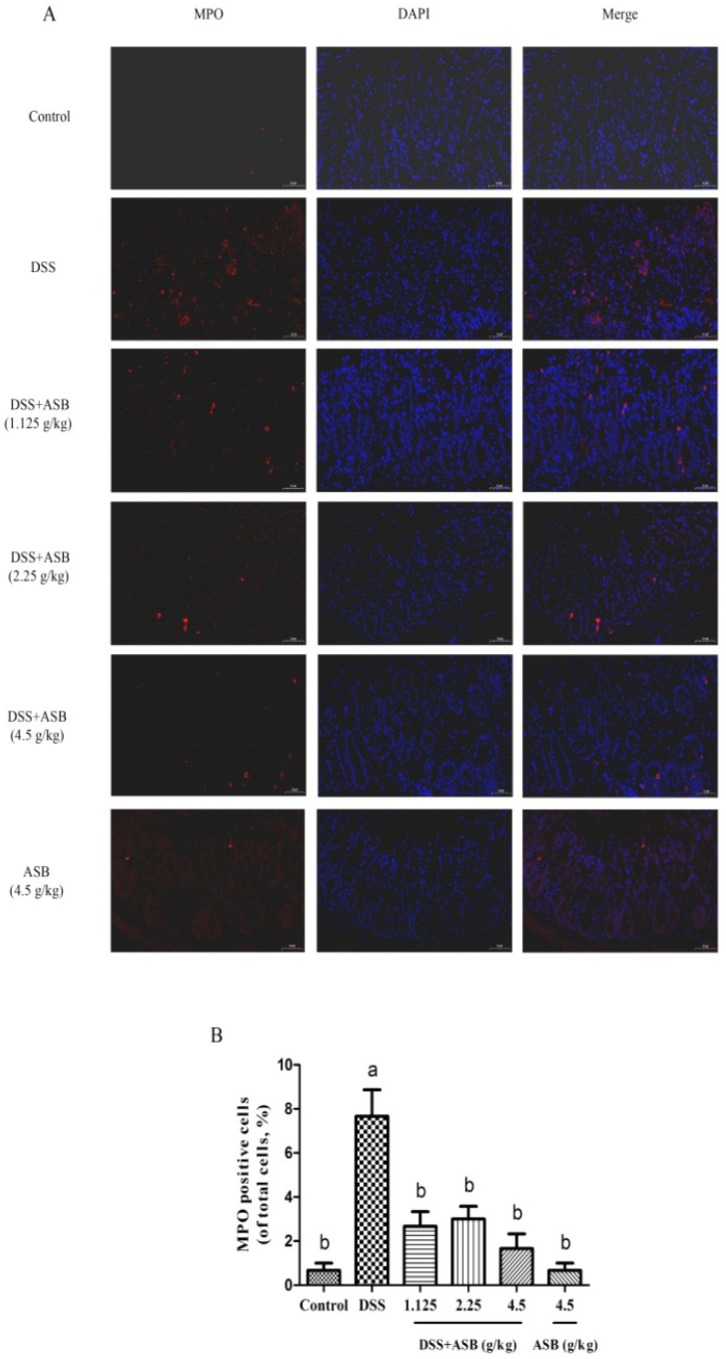
Effect of ASB on the levels of an inflammatory factor in the colon. Expression of MPO in colonic tissue was assessed by immunofluorescence (scale bar: 50 µm) (**A**) and MPO-positive cells quantification (**B**). Results were expressed as mean ± SD (*n* = 3). The expression levels of MPO marked by the same letter are not significantly different. Significance is represented as *p* < 0.05.

Similar to the colon histopathology scores, the expression level of colonic myeloperoxidase (MPO) was greatly up-regulated in the DSS group, while the MPO activities in the ASB-treatment groups were markedly reduced (Figure 4A). As shown in Figure 4B, less MPO-positive cells were detected in the high dosage reference group, which was similar to the control group.

### 2.7. ASB Suppressed the Production of Pro-Inflammatory Mediators in the Impaired Colon

**Figure 5:**
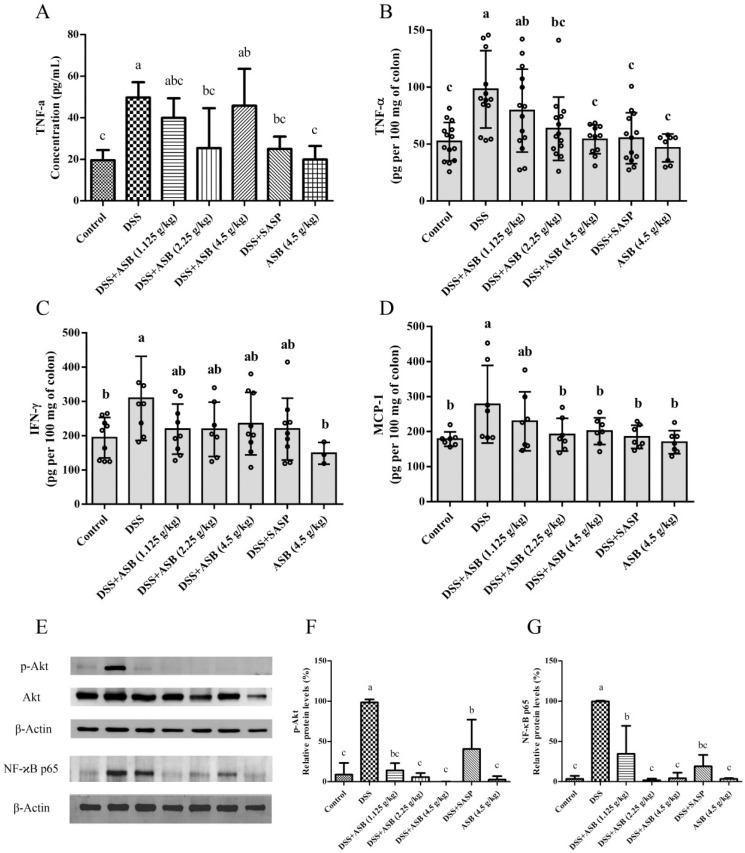
(**A**) Effects of ASB on the production of cytokines TNF-α in the serum of mice with DSS-induced colitis (*n* = 3). Effects of ASB on the production of cytokines (**B**) TNF-α (C: control, *n* = 14; D: DSS model, *n* = 14; L: DSS+ASB low dosage, *n* = 14; M: DSS+ASB medium dosage, *n* = 14; H: DSS+ASB high dosage, *n* = 11; P: SASP, *n* = 13; T: ASB high dosage reference, *n* = 8), (**C**) IFN-γ (C: control, *n* = 9; D: DSS model, *n* = 8; L: DSS+ASB low dosage, *n* = 9; M: DSS+ASB medium dosage, *n* = 7; H: DSS+ASB high dosage, *n* = 9; P: SASP, *n* = 9; T: ASB high dosage reference, *n* = 3), and (**D**) MCP-1 (*n* = 7) in the culture supernatants of colonic tissue of mice with DSS-induced colitis. ASB ameliorates DSS-induced colitis via the TLR4-linked NF-κB signaling pathway. (**E**) Protein levels of p-Akt and NF-κB in the colon were assessed by Western blotting. (**F**,**G**) Relative protein levels of p-Akt/β-Actin and NF-κB/β-Actin (*n* = 3). Results were expressed as mean ± SD. Parameters marked by the same letter are not significantly different. Significance is represented as *p* < 0.05.

As shown in Figure 5A, treatment with SASP markedly inhibited the secretion of TNF-α (25.06 ± 5.88 pg/mL) in serum as compared to the secretion in the DSS model group (49.77 ± 7.34 pg/mL). ASB treatments with high, medium, and low dosage also inhibited the production of TNF-α (45.84 ± 17.70 pg/mL, 25.45 ± 19.16 pg/mL, 39.96 ± 9.44 pg/mL, respectively) in serum in comparison with the DSS group, similar to the SASP-treated group. Results also indicated that the expression level of TNF-α (19.96 ± 6.48 pg/mL) in serum presents normal in the high dosage reference group, similar to the expression level in the control group (19.57 ± 4.90 pg/mL). Moreover, results in Figure 5B–D showed that ASB treatments and SASP treatment could greatly down-regulate the expression level of pro-inflammatory mediators TNF-α, IFN-γ, and MCP-1 in colonic tissue. TNF-α production (54.22 ± 12.61 pg/mL, 63.51 ± 27.83 pg/mL, 79.43 ± 36.41 pg/mL, respectively) in colonic tissue was significantly suppressed upon ASB treatments with high, medium and low dosage as compared to the production (98.15 ± 33.93 pg/mL) in the DSS group (Figure 5B). As shown in Figure 5C, DSS-induced colitis markedly increased the production of IFN-γ (309.23 ± 122.83 pg/mL) in colonic tissue, while the production was suppressed upon ASB treatments with high, medium and low dosage (235.22 ± 91.03 pg/mL, 219.13 ± 79.14 pg/mL, 219.52 ± 72.98 pg/mL, respectively). Results in Figure 5D also indicated that ASB treatments with high, medium and low dosage could significantly suppress MCP-1 production (201.57 ± 38.34 pg/mL, 191.57 ± 47.17 pg/mL, 229.67 ± 84.42 pg/mL, respectively) in colonic tissue as compared to the production in DSS-induced colitis (278.24 ± 110.97 pg/mL). Meanwhile, results also indicated that the expression level of TNF-α, IFN-γ, and MCP-1 in colonic tissue were normal in the high dosage reference group, similar to the control group.

### 2.8. ASB Improved UC through TLR4 Signaling Inhibition

As shown in Figure 5E–G, the protein levels of NF-κB and p-Akt in colonic tissues were increased in DSS-induced colitis, in comparison with the control group. However, results showed that ASB treatments could significantly down-regulate the protein expression levels of NF-κB and p-Akt in the inflamed colon tissues, similar to the control group. 

### 2.9. ASB Down-Regulated the Expression Levels of Inflammatory Mediators in LPS-Activated Macrophages through TLR4 Signaling Inhibition

**Figure 6:**
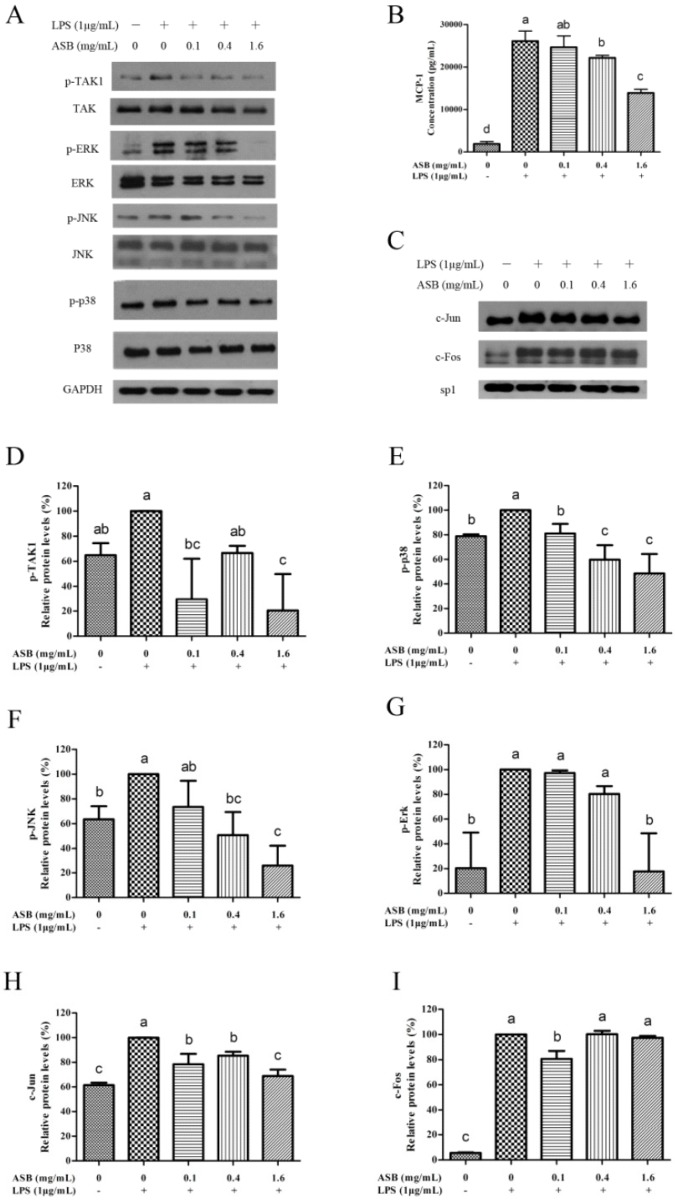
Effects of ASB on the phosphorylation of protein levels on AP-1 pathways in LPS-induced macrophages (**A**), and relative protein levels of p-TAK1 (**D**), p-p38 (**E**), p-JNK (**F**), and p-Erk (**G**). Expression levels of nuclear proteins of transcription factors NF-κB and AP-1 regulated upon ASB treatment (**C**), and relative protein levels of c-Jun (**H**) and c-Fos (**I**). (**B**) Effects of ASB on the secretion of MCP-1 in the culture of LPS-induced macrophages. Results were expressed as mean ± SD. Relative protein levels of each column marked by the same letter are not significantly different. Significance is represented as *p* < 0.05.

As shown in Figure 6C, the production of MCP-1 was markedly increased in the culture of LPS-activated macrophages compared to the control, while the secretion of MCP-1 was significantly suppressed upon ASB treatment in a dose-dependent manner. Moreover, significant (*p* < 0.05) differences were also found in the phosphorylation of TAK1, ERK, JNK, and p38 in TLR4 signaling, except for the protein expression level of ERK at 0.1 mg/mL (Figure 6A,D–G). Due to the phosphorylation of ERK, JNK, and p38, the protein expression levels were down-regulated in the LPS-activated macrophages with dose-dependency upon ASB treatments. Meanwhile, one of the AP-1 components (c-Jun) also significantly reduced the corresponding nuclear localization in LPS-activated macrophages upon ASB treatments (Figure 6C,H,I).

## 3. Discussion

The DSS-induced colitis in C57BL/6 mice showed many similarities in appearance to ulcerative colitis, together with several pathophysiological and morphological features, such as weight loss, shortened colon length, production of inflammatory mediators, crypt damage, and focal inflammation infiltration. Sulfasalazine (SASP), a drug which has been used for treating inflammatory bowel disease (IBD) for decades, is commonly used as a positive control in colitis study [30,31,32]. The results indicated that ASB treatments showed many similarities to SASP-positive control treatment in treating colitis. In this study, the DSS group demonstrated anemia (lower RBC levels, HGB, HCT, MCV, and PDW), which was significant (*p* < 0.05) as compared to the control group, and which is in agreement with the report by Larrosa et al. (2009) [33]. Meanwhile, results showed that ASB treatments could significantly ameliorate these changes (Table 1). Moreover, body weight, feed consumption, colon length, and DAI were commonly applied as the indicators for evaluating the disease severity of DSS-induced colitis [10,11]. Many researchers have shown that the DSS group could markedly shorten the colon length, lower the body weight, and reduce feed consumption, which is similar to the result in this study [11,34]. Treatments with ASB could significantly prevent colon length shortening, reduce food consumption, and help in losing body weight. The current result (Figure 2B,H) indicated that ASB treatments could reduce rectal bleeding and ameliorate colitis in mice with DSS-induced colitis, similar to the report by Markovic et al. (2016) and Yan et al. (2018) [14,35]. Intestinal permeability was performed to evaluate the barrier function, as it is an important indicator for assessing colitis. Results of various studies indicated that the increase in gut permeability was linked with greater susceptibility to colitis [36,37]. The FITC-Dextran assay was a typical method for the in vivo assessment of intestinal permeability [36,37]. Consistent with the results in colon length and body weight, results showed that ASB could significantly reduce intestinal permeability. Collectively, the results showed that DSS successfully induced colitis, similar to the previous findings. In addition, the results suggest that ASB administration might ameliorate UC. 

For further confirmation, histopathology was studied for assessing the degree of colonic tissue damage and neutrophil infiltration. With this context, previous work by Zhu et al. (2017) and Yan et al. (2018) have already indicated that DSS could cause colonic tissue damage, including inflammatory cell infiltration and crypt damage, leading to higher histological score, while such a change could be improved upon suitable treatments [11,14]. The current study showed that ASB could significantly reduce the colonic tissue damage and lead to a lower histological score as compared to the DSS group. Moreover, myeloperoxidase (MPO) is a critical marker for neutrophils, correlating with the extent of neutrophil infiltration [34,38]. Therefore, it is very meaningful to detect the expression level of MPO in colonic tissue to evaluate the degree of colonic damage. Previous studies have already reported that neutrophil infiltration into injured colonic tissue could accelerate the damage of colonic tissue by enzyme MPO [8,34,38]. The current results showed that the expression level of MPO in inflamed tissue could be down-regulated upon ASB treatment (Figure 4A,B). Together, results suggested that ASB treatments with different dosages showed dietary efficacy in ulcerative colitis (UC) amelioration. Based on the evaluation of DAI (Figure 2H), ASB treatment at the dosage of 2.25 g/kg b.w. (the human equivalent dose (HED): 400 g/60 kg) could be treated as the suggested dose for sea bass consumption. Based on the current study, it was clearly indicated that ASB possessed potential therapeutic efficacy against DSS-induced colitis. 

Inflammation plays a vital role in DSS-induced colitis. TLR4 signaling, one of the important mechanisms for inflammation, is a key receptor for commensal recognition in gut innate immunity [12,39,40]. TLR4 signaling was the subject of therapeutics (target inhibition) in ulcerative colitis (UC) [12,13,41]. Moreover, AP-1 and NF-κB were treated as the critical and classical pathways in TLR4 signaling [42,43]. Many researchers have already elucidated that over-expression of TLR4-linked AP-1 or NF-κB activation is typical in inflamed colonic tissue [13,14]. Therefore, in vivo and in vitro studies on the efficacy of ASB treatment in inflammation through TLR4 signaling have been studied. The present in vivo and in vitro studies showed that the activation of TLR4 signaling was up-regulated in the DSS-induced colitis and LPS-activated macrophages. However, the up-regulation of TLR4 signaling was markedly inhibited upon ASB treatments. The results indicated that inflammatory mediators in DSS-induced colitis could be inhibited upon ASB treatments, similar to the previous researches [36,44,45]. The in vitro study also indicated that ASB could suppress the inflammatory mediators in LPS-activated macrophage through inhibiting TLR4-linked AP-1 activation. According to the in vivo and in vitro studies, current results demonstrated that ASB treatments ameliorate the intestinal inflammation in the gut and are correlated with the suppression of the activation of TLR4 signaling. 

## 4. Materials and Methods

### 4.1. Materials and Reagents

Lipopolysaccharide (LPS, purified from *Escherichia coli* O55: B5) and bovine serum albumin were obtained from Sigma Chemical Co. (St. Louis, MO, USA). RAW 264.7 cell line (ATCC no.: TIB-71) derived from murine macrophages was purchased from ATCC (Rockville, MD, USA). TNF-α (D2D4), Akt (11E7), phospho-Akt (Ser473), and NF-κB (p65) were obtained from Cell Signaling Technology (Boston, MA, USA). Myeloperoxidase (MPO) was obtained from Abcam (Cambridge, UK). Other antibodies in NF-κB pathways were purchased from Santa Cruz Biotechnology (Santa Cruz, CA, USA). ELISA kits for the determination of the cytokines TNF-ɑ, MCP-1, and IFN-γ were purchased from Invitrogen (Carlsbad, CA, USA). All other chemicals were of analytical grade. 

### 4.2. Sea Bass Materials and Preparation of Aqueous Extract of Sea Bass

Baijiao sea bass (*Lateolabrax japonicus*) was collected from Estuarine Fisheries Research Institute in Zhuhai, Guangdong Province, China. Aqueous extract of sea bass (ASB) was prepared via steaming, size reduction, sonication, and freeze-drying [46]. Briefly, the edible parts of sea bass were dissected and steamed for 10 min. After steaming, fish bones were removed and the edible parts were homogenized. Then, the homogenized meat was weighed in the beaker, and distilled water (ratio of solid to liquid at 1:5) was added, and then extracted by ultrasonication for 2 h at 90~100 °C. It was followed by freezing at −80 °C and freeze-drying via vacuum freeze—drier (FreeZone Benchtop, Labconco Company, Kansas City, MO, USA). The final products (the aqueous extract of sea bass, ASB) were weighted and stored in −80 °C refrigerator for further analysis.

### 4.3. Characterization of the Aqueous Extract of Sea Bass (ASB)

Crude protein content was determined using the Kjeldahl method based on the procedure described by Kirk (1950) [47]. The molecular weight of the protein fractions of ASB was analyzed by SDS-PAGE (sodium dodecyl sulphate-polyacrylamide gel electrophoresis) based on the method of Jambrak et al. (2014) with some modifications [48]. The amino acids content of ASB from each batch was analyzed using Biochrom 30 amino acid analyzer (DKSH management Ltd., Shanghai, China).

### 4.4. Animals

Male C57BL/6 mice (8 weeks, ~22 g) were purchased from SPF (Beijing) Biotechnology Co., Ltd. Animal procedures were approved by the Ethics Committee of Hong Kong Baptist University with an ethical code (REC/18-19/0008, 18 October 2018, committee on the use of human & animal subjects in teaching and research, Hong Kong Baptist University). All animal treatments complied with the “Guide for the Care and Use of Laboratory Animals” published by the National Institutes of Health (NIH).

### 4.5. Establishment of Ulcerative Colitis (UC) Model

Ulcerative colitis was induced by the oral administration of 1.5% (*w*/*v*) DSS (relative molecular mass 36–50 kDa; MP Biomedicals) dissolved in drinking water for 7 days according to Heinsbroek et al. (2015) and Zhu et al. (2017) with some modifications [9,11]. Mice were arbitrarily allocated into seven groups: control group (DDI water, *n* = 14 per group), DSS model group (*n* = 14 per group), three ASB treated groups (low-dosage: 1.125 g/kg b.w./day; mid-dosage: 2.25 g/kg b.w./day; high-dosage: 4.5 g/kg b.w./day, *n* = 14 per group), high-dosage reference group (high-dosage: 4.5 g/kg b.w./day, *n* = 8 per group) and sulfasalazine (SASP)-positive control group (SASP, 50 mg/kg b.w./day, *n* = 14 per group). ASB, SASP, and DDI water were given orally by gavage, once daily for 9 days. Meanwhile, drinking water was replaced with 1.5% (*w*/*v*) DSS solution in the DSS model group, three ASB-treated groups, and SASP-treated group for the first 7 days. Control group and high dosage reference groups received drinking water without DSS throughout the experiment period. 

### 4.6. Intestinal Permeability In Vivo

The measurement of intestinal permeability towards FITC-Dextran (4 kDa, Catalog#4009, Chondrex, Redmond, WA, USA) was performed according to the manufacturer’s protocol and Cani et al. (2009) [36]. Briefly, mice that had fasted for 4 h were given FITC-Dextran by oral gavage (500 mg/kg body weight, 25 mg/mL). After maintaining fasting conditions for 3 h, blood was collected from the orbital venous plexus. Then, blood was centrifuged at 14,000× *g* for 5 min at 4 °C. Plasma was diluted in an equal volume of PBS and read on a fluorescent plate reader at an excitation wavelength of 485 nm and an emission wavelength of 520 nm. A standard curve was prepared by making dilutions of the stock FITC-dextran in normal mouse plasma diluted with PBS.

### 4.7. Disease Activity Index (DAI)

The characterization of colitis symptoms was monitored by the underlying body weight, stool consistency, stool color, and occult bleeding. The DAI was evaluated based on the scoring methods of Chen et al. (2017) and Yan et al. (2018) with some modifications [10,14]. Weight loss was calculated based on the difference in body weight of mice between day 0 and testing day. Stool consistency and stool color were monitored base on the fecal pellet formation and visible blood by visual identity. Occult blood was analyzed using a fecal occult blood test kit (Nanjing Jiancheng Bioengineering Institute, Nanjing, China). The DAI values were conducted by the sum of the score from weight loss, stool consistency, stool color, and occult bleeding. 

### 4.8. Histology and Immunofluorescence

At the end of the experiment, all mice were euthanized. The colon was dissected and the length between ileo-cecal junction and anal verge was measured. The colonic tissues were fixed in 10% formalin solution overnight and then processed, embedded in paraffin and cut into 4-µm-thick sections. Histopathological examination was conducted by H & E staining according to the manufacturer’s instructions. Images were obtained by Nikon Eclipse C1 microscopy (Japan). The histological scoring system (Figure 3B) was described by Xiao et al. (2013) and Winter et al. (2013), with some modifications [34,49]. To quantify the inflammatory responses in colonic tissue, the expression of MPO was detected by immunofluorescence (IF) analysis. The positive expression levels in IF images were processed and quantified using Image-Pro Plus 6.0. 

### 4.9. Cell Culture

RAW 264.7 cells were cultured in DMEM containing 10% FBS and 1% antibiotics (penicillin-streptomycin) at 37 °C under a humidified atmosphere of 95% air and 5% CO_2_ by referring to the method described previously [50].

### 4.10. Western Blotting

For the immunoblot analysis of TLR4, cytoplasmic protein extraction method and analysis were applied, based on the method of Lai et al. (2017) with slight modifications [51]. RAW 264.7 macrophages were grown to confluence in 6-well plates for overnight adhesion and subsequently treated with various concentrations (0.1, 0.4, and 1.6 mg/mL) of ASB for 1 h before LPS (1 µg/mL) stimulation. After 24 h incubation, the cells were collected for Western blotting. The cells were washed with ice-cold PBS twice and then incubated with lysis buffer for 30 min on ice. Supernatants were collected by centrifugation at 13,523× *g* for 15 min. For nuclear protein extraction, the method of Cheng et al. (2015) was applied, with slight modifications [28]. The collected cells were washed with PBS, and then, hypotonic buffer (15 nM MgCl_2_, 10 mM KCl, and 20 nM Tris-HCl (pH 7.9)) was added for extraction for 15 min on ice. Then, 12 µL NP-40 (10%, *v/v*) was added for another 10 min. The supernatants were collected as cytoplasmic extracts by centrifugation at 15,777× *g* at 4 °C for 1 min. The remaining pellets were washed with 100 µL hypotonic buffer and then suspended in a high salt buffer (0.2 mM EDTA, 1.5 mM MgCl_2_, 0.42 M NaCl, 25% glycerol, and 20 mM Tris-HCl, pH 7.9) on ice for 30 min. The nuclear protein was obtained via centrifugation at 15,777× *g* for 10 min at 4 °C. For the in vivo experiment, colonic tissues were homogenized in RIPA lysis buffer containing proteinase and phosphatase inhibitor and incubated for 20 min on ice. Supernatants were collected by centrifugation at 13,523× *g* for 60 min. The concentrations of the extracted proteins were measured using a BSA protein assay. An equal amount of extracted proteins (20–40 µg) was loaded onto the prepared gel (8~12% (*w/v*)) for Western blotting. The membranes were blocked with milk for 1 h and then washed with TBST for 10 min. Primary antibodies were diluted in 3% BSA and overnight cultured with membranes at 4 °C for shaking. Subsequently, membranes were washed with TBST for 20 min three times and then incubated with secondary antibody at room temperature for 1 h. Finally, the membranes were visualized by soaking in a chemiluminescent substrate and then exposed to obtain the signal. Band images were obtained using EPSON scanner, and band densities were analyzed using the Image J software (BioTechniques, New, York, NY, USA). 

### 4.11. Enzyme-Linked Immunosorbent Assay (ELISA) Analysis

The secretion of cytokine (MCP-1) (eBioscience, San Diego, CA, USA) in the culture of LPS-stimulated macrophages was measured using ELISA kits by following the manufacturer’s instruction. Additionally, the production of cytokines TNF-α in serum and MCP-1, TNF-α, and IFN-γ in the culture supernatants of colonic tissue of mice upon treatments (eBioscience) were quantified using ELISA kits according to the manufacturer’s instructions.

### 4.12. Statistical Analysis

Analyses were performed in triplicates, and results were expressed as mean ± SD. For multiple group comparisons, one-way analysis of variance (ANOVA) was conducted by Dunnett’s post hoc test and applied for determining the significance (*p* < 0.05) differences. Statistical analyses were performed using Microsoft 2016 package and SPSS (SPSS 17.0, SPSS Inc., Chicago, IL, USA).

## 5. Conclusions

The current results clearly indicate that ASB possesses potential anti-inflammation therapeutic efficacy through inhibiting the activation of TLR4 signaling against DSS-induced colitis and LPS-activated macrophages. According to the in vivo and in vitro studies, the activation of TLR4 signaling was significantly inhibited upon ASB treatments. The production of pro-inflammatory cytokines in inflamed models was markedly reduced upon ASB treatments. Results also indicated that ASB could significantly ameliorate several pathophysiological and morphological features in DSS-induced colitis. The current work illustrated that ASB demonstrated an inhibitory efficacy on TLR4 signaling activation, and thus, could be a promising candidate for treating UC. In addition, it also establishes a pharmacological basis for the folk use of sea bass. 

## Figures and Tables

**Table 1:** Hematological parameters in mice.

Hematological Parameters	Control	DSS	DSS+ASB (1.125 g/kg)	DSS+ASB (2.25 g/kg)	DSS+ASB (4.5 g/kg)	DSS+SASP	NVR
RBC (10^12^/L)	7.39 ± 0.55 **a**	5.37 ± 1.25 **c**	6.67 ± 1.15 **ab**	6.23 ± 0.64 **bc**	6.01 ± 0.90 **bc**	6.07 ± 0.56 **bc**	6.68~8.28
HGB (g/L)	115 ± 8 **a**	83 ± 20 **c**	101 ± 17 **ab**	95 ± 10 **bc**	93 ± 14 **bc**	90 ± 8 **bc**	106~129
HCT (%)	37.2 ± 2.6 **a**	26.3 ± 6.1 **c**	32.8 ± 5.7 **ab**	30.5 ± 3.2 **bc**	29.3 ± 4.5 **bc**	29.3 ± 2.5 **bc**	33.9~41.8
MCV (fL)	50.4 ± 0.8 **a**	49.0 ± 1.0 **b**	49.2 ± 0.7 **b**	48.9 ± 0.5 **b**	48.8 ± 0.5 **b**	48.4 ± 1.7 **b**	49.2~51.3
MCH (pg)	15.5 ± 0.2 **a**	15.4 ± 0.2 **ab**	15.2 ± 0.1 **b**	15.3 ± 0.1 **ab**	15.5 ± 0.2 **a**	14.9 ± 0.6 **c**	15.2~15.9
MCHC (g/L)	308 ± 4 **bc**	314 ± 8 **ab**	309 ± 4 **bc**	314 ± 5 **ab**	318 ± 5 **a**	308 ± 4 **bc**	299~313
PDW	14.8 ± 0.1 **a**	14.3 ± 0.2 **c**	14.6 ± 0.2 **b**	14.5 ± 0.2 **b**	14.5 ± 0.2 **b**	14.4 ± 0.1 **bc**	14.7~14.9
WBC (10^9^/L)	3.76 ± 0.65 **ab**	3.30 ± 0.87 **b**	5.14 ± 1.10 **a**	4.07 ± 2.34 **ab**	2.59 ± 0.95 **b**	4.02 ± 1.29 **ab**	2.53~4.62
Neu# (10^9^/L)	2.08 ± 0.81 **abc**	1.52 ± 0.34 **bc**	2.84 ± 0.61 **a**	2.29 ± 1.91 **ab**	1.15 ± 0.52 **c**	1.86 ± 0.63 **abc**	0.47~3.01
Lymph# (10^9^/L)	1.68 ± 0.35 **ab**	1.78 ± 0.61 **ab**	2.29 ± 0.62 **a**	1.77 ± 0.60 **ab**	1.43 ± 0.64 **b**	2.16 ± 0.72 **a**	1.23~2.31
Mon# (10^9^/L)	0 ± 0 **b**	0 ± 0 **b**	0.01 ± 0.01 **a**	0 ± 0 **b**	0 ± 0 **b**	0 ± 0 **b**	0
Eos# (10^9^/L)	0 ± 0.01 **a**	0 ± 0 **a**	0 ± 0 **a**	0.01 ± 0.01 **a**	0 ± 0 **a**	0 ± 0 **a**	0~0.02
Bas# (10^9^/L)	0 ± 0 **b**	0 ± 0 **b**	0 ± 0 **b**	0 ± 0 **b**	0.01 ± 0.01 **a**	0 ± 0 **b**	0
Neu% (%)	53.3 ± 16.5 **a**	46.8 ± 6.3 **a**	55.5 ± 6.7 **a**	51.6 ± 12.7 **a**	45.2 ± 13.2 **a**	46.2 ± 4.9 **a**	39.6~66.1
Lymph% (%)	46.5 ± 16.4 **a**	53.1 ± 6.3 **a**	44.2 ± 6.8 **a**	48.0 ± 12.4 **a**	54.1 ± 13.2 **a**	53.7 ± 5.0 **a**	33.7~81.3
Mon% (%)	0 ± 0 **b**	0.04 ± 0.1 **ab**	0.2 ± 0.2 **a**	0.1 ± 0.1 **ab**	0.1 ± 0.2 **ab**	0.1 ± 0.1 **ab**	0
Eos% (%)	0.2 ± 0.2 **a**	0 ± 0 **a**	0.1 ± 0.1 **a**	0.2 ± 0.4 **a**	0.2 ± 0.2 **a**	0 ± 0 **a**	0~0.7
Bas% (%)	0 ± 0.1 **b**	0.1 ± 0.1 **b**	0 ± 0.1 **b**	0.2 ± 0.2 **b**	0.4 ± 0.4 **a**	0 ± 0 **b**	0~0.2
RDW-CV (%)	13.3 ± 0.8 **b**	13.6 ± 2.6 **b**	13.6 ± 1.3 **b**	13.7 ± 1.3 **b**	13.1 ± 0.6 **b**	17.2 ± 1.6 **a**	12.6~15.1
PLT (10^9^/L)	611 ± 53 **a**	576 ± 146 **a**	681 ± 122 **a**	631 ± 94 **a**	569 ± 128 **a**	688 ± 91 **a**	526~662
MPV (fL)	5.3 ± 0.2 **a**	5.3 ± 0.2 **a**	5.3 ± 0.2 **a**	5.4 ± 0.2 **a**	5.3 ± 0.1 **a**	5.3 ± 0.1 **a**	5.2~5.7
PCT (%)	0.325 ± 0.032 **a**	0.304 ± 0.072 **a**	0.359 ± 0.067 **a**	0.339 ± 0.058 **a**	0.299 ± 0.065 **a**	0.363 ± 0.048 **a**	0.274~0.362

Results were expressed as mean ± SD, *n* = 8 independent experiments. WBC, white blood cell; Neu#, neutrophil values; Lymph#, lymphocyte values; Mon#, monocyte values; Eos#, eosinophil values; Bas#, basophil values; Neu%, Lymph%, Mon%, Eos% and Bas%, percentages of corresponding cell over white blood cell; RBC, red blood cell; HGB, hemoglobin; HCT, hematocrit; MCV, mean corpuscular volume; MCH, mean corpuscular hemoglobin; MCHC, mean corpuscular hemoglobin concentration; RDW-CV, coefficient of variation of erythrocyte distribution width; PLT, platelets; MPV, mean platelet value; PDW, platelet distribution width; PCT, procalcitonin. Hematological inflammatory parameters of each row marked by the same letter are not significantly different. Significance is represented as *p* < 0.05.

## Supplementary Materials

Supplementary materials can be found at https://www.mdpi.com/1422-0067/20/12/2907/s1.

## Abbreviations

ASB	Aqueous extract of sea bass
DSS	Dextran sulphate sodium
ELISA	Enzyme-linked immunosorbent assay
IBD	Inflammatory bowel disease
IFN-γ	Interferon gamma
IF	Immunofluorescence
LPS	Lipopolysaccharide
MPO	Myeloperoxidase
MCP-1	Monocyte chemoattractant protein-1
SASP	Sulfasalazine
TLR	Toll-like receptor
TNF-α	Tumor necrosis factor alpha
UC	Ulcerative colitis
