# Supplementary material for: Dietary Supplementation with Sea Bass (Lateolabrax maculatus) Ameliorates Ulcerative Colitis and Inflammation in Macrophages through Inhibiting Toll-Like Receptor 4-Linked Pathways
Source: Int J Mol Sci. 2019 Jun 14;20(12):2907. doi: 10.3390/ijms20122907 (PMC6628281; doi:10.3390/ijms20122907)
Supplement: Supplementary file 1 [file ijms-20-02907-s001.pdf]

Supplementary Materials for online publication

Title: Dietary supplementation with sea bass (*Lateolabrax maculatus*) ameliorates ulcerative colitis and inflammation in macrophages through inhibiting toll-like receptor 4-linked pathways

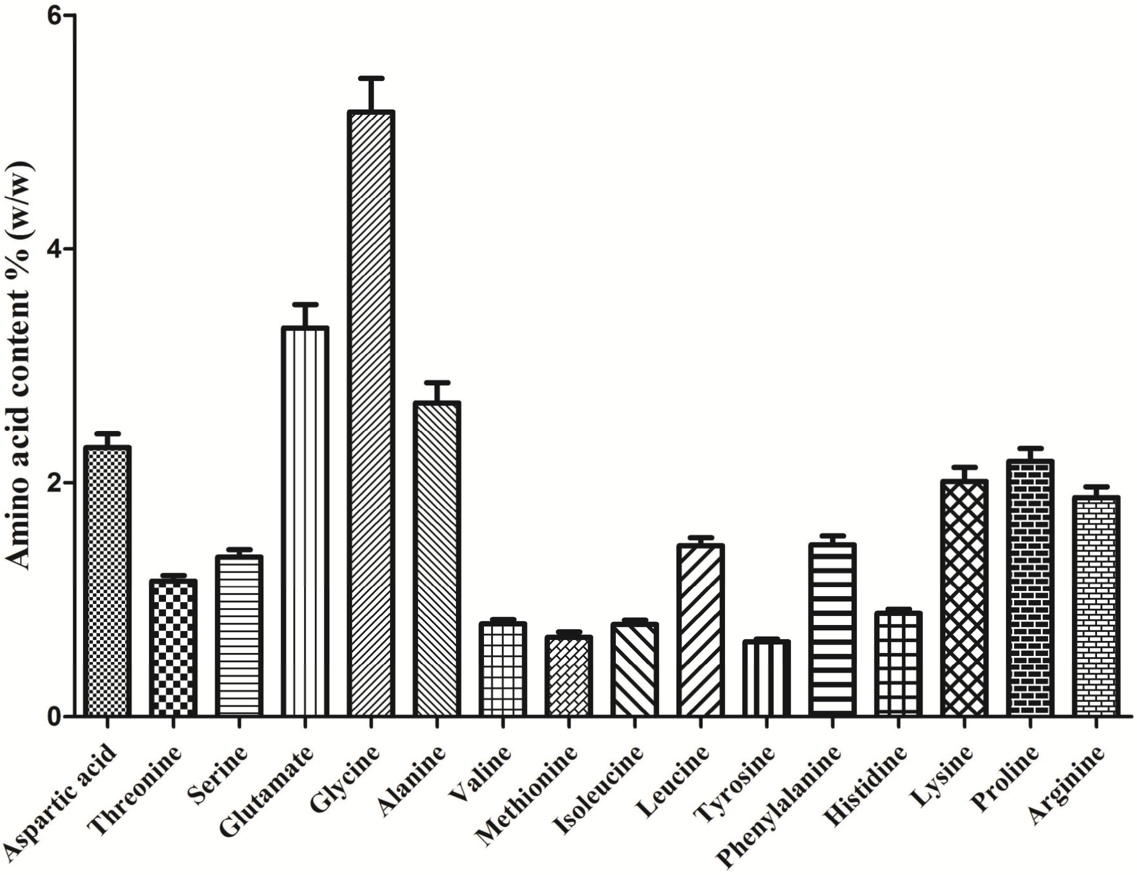

Supplementary Figure 1. The amounts of 16 kinds of amino acids in samples. The values were presented as mean ± SD of three independent experiments.

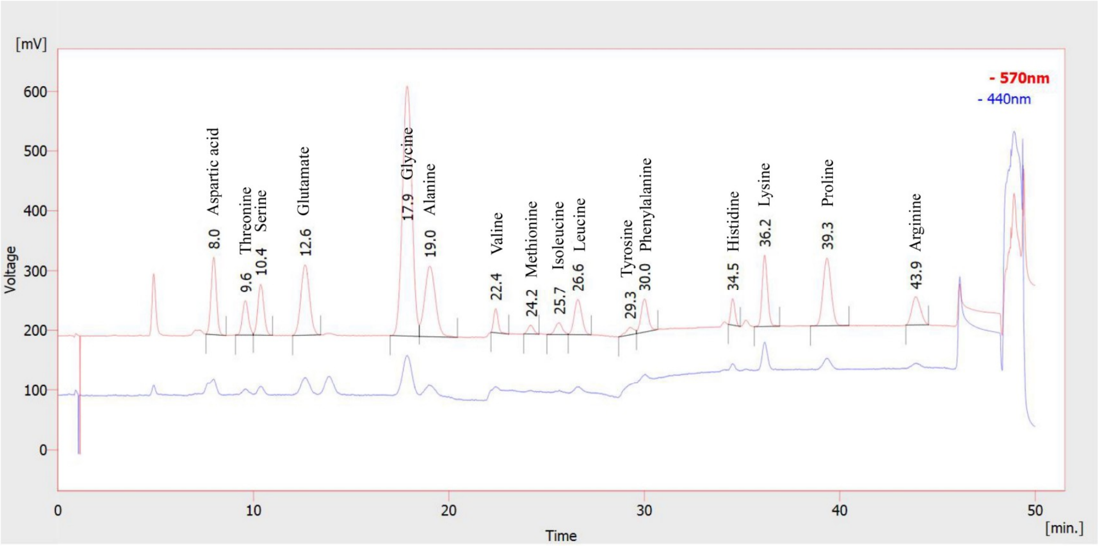

Supplementary Figure 2. Typical chromatogram of 16 kinds of amino acids in sample.
